# Grocery Delivery of Healthy Foods to Pregnant Young Women With Low Incomes: Feasibility and Acceptability Mixed Methods Study

**DOI:** 10.2196/21602

**Published:** 2020-12-24

**Authors:** Ione Locher, Marika Waselewski, Kendrin Sonneville, Ken Resnicow, Tammy Chang

**Affiliations:** 1 University of Michigan Ann Arbor, MI United States

**Keywords:** pregnancy, adolescent, young adult, female, gestational weight gain, diet, food preferences, text messaging, feasibility studies

## Abstract

**Background:**

Poor maternal diets increase the risk of excess gestational weight gain which can contribute to serious intergenerational morbidity for both the mother and infant. Pregnant young women with low incomes have disproportionately high rates of inadequate fruit and vegetable consumption as well as excess weight gains during pregnancy.

**Objective:**

Our aim was to describe the feasibility and acceptability of Special Delivery, a longitudinal nutrition intervention that delivers healthy foods to pregnant youth (aged 14-24 years) with low incomes.

**Methods:**

The Special Delivery pilot study, conducted in Michigan, enrolled pregnant young women with low incomes. Study participants were sent twice-monthly grocery deliveries consisting of US $35 worth of healthy foods, primarily fruits and vegetables. Between grocery deliveries, participants received daily SMS text message prompts to confirm receipt of delivery and document diet and weight. Program feasibility was assessed by the number of grocery orders placed, delivered, and confirmed by participants. Qualitative interviews and SMS text message data were used to determine acceptability by assessing participants’ perspectives on grocery delivery, participants’ perspectives on dietary impact of the program, and foods consumed by participants.

**Results:**

A total of 27 participants were enrolled in the pilot study. The mean age was 20.3 years (SD 2.0), and 59.3% (16/27) were African American or Black. During the pilot, 263 deliveries were sent with 98.5% (259/263) successful deliveries and 89.4% (235/263) deliveries confirmed by participants. Participants reported that grocery delivery was convenient; that delivered foods were high quality; and that the program improved their diet, increased access to healthy foods, and promoted healthy habits during pregnancy.

**Conclusions:**

A grocery delivery–based weight gain and nutrition intervention is both feasible and acceptable among low-income pregnant youth. Grocery deliveries were successfully completed and participants were willing and able to receive grocery deliveries, eat the healthy foods that were delivered, and communicate via SMS text message with study coordinators. The Special Delivery program warrants further evaluation for efficacy in promoting healthy weight gain for low-income youth during pregnancy.

## Introduction

Pregnancy is a crucial time for women to make healthy dietary choices. Maternal consumption of fruit and vegetables is associated with healthy pregnancy weight gain and reduced risk of poor pregnancy outcomes such as miscarriage, gestational hypertension, and gestational diabetes [1-3]. Meanwhile, dietary patterns that result in excess gestational weight gain (defined as weight gain above the National Academy of Medicine gestational weight gain guidelines [4]) can have long-term consequences on lifetime weight gain and health outcomes for pregnant woman and their children [5]. Excess weight gain in pregnancy can also lead to complications during birth and permanently impacts fetal genetic programming, which determines risk for chronic disease among infants [6-9]. Adolescent pregnant women are at significantly higher risk of excess gestational weight compared to older women [4,10,11]. Women with low incomes are at additional risk for both low fruit and vegetable consumption and excess gestational weight gain [12,13].

The Special Supplemental Nutrition Program for Women, Infants, and Children (WIC) is a federally (United States) funded program that provides financial support for the purchase of healthy foods to pregnant women with low incomes and their children, to encourage healthy eating during and after pregnancy. However, many young mothers are unable to adequately access these healthy foods due to logistical barriers such as lack of transportation, little experience in grocery shopping, and difficulty navigating WIC benefit redemption requirements [14-17]. As convenience and availability of healthy foods are among the most powerful factors driving weight gain and diet behavior [18-20], innovative approaches that minimize these logistical barriers should be examined. Grocery delivery represents a well-established, inexpensive, and convenient service that can significantly increase availability of healthy foods [21]. Grocery delivery has already been demonstrated to improve access to healthy food in underresourced areas, such as urban food deserts [22]. In addition to providing convenient access to healthy foods, grocery delivery of healthy foods has been shown to improve diet quality [21,23,24]. Home delivery of non sugar-sweetened beverages to adolescents was associated with significant reductions in adolescents’ intake of sugar-sweetened beverages, and the impact persisted 2 years after the intervention [25].

No studies to date have assessed the impact of providing home grocery delivery to pregnant young women. To address the critical need for increased access to healthy food for pregnant adolescents with low incomes, we developed an intervention that uses twice-monthly home delivery of groceries to provide healthy food options to participating pregnant young women with low incomes in southeast Michigan. In this paper, we describe our pilot study to assess feasibility and acceptability of this intervention.

## Methods

### Overview

**Figure 1:**
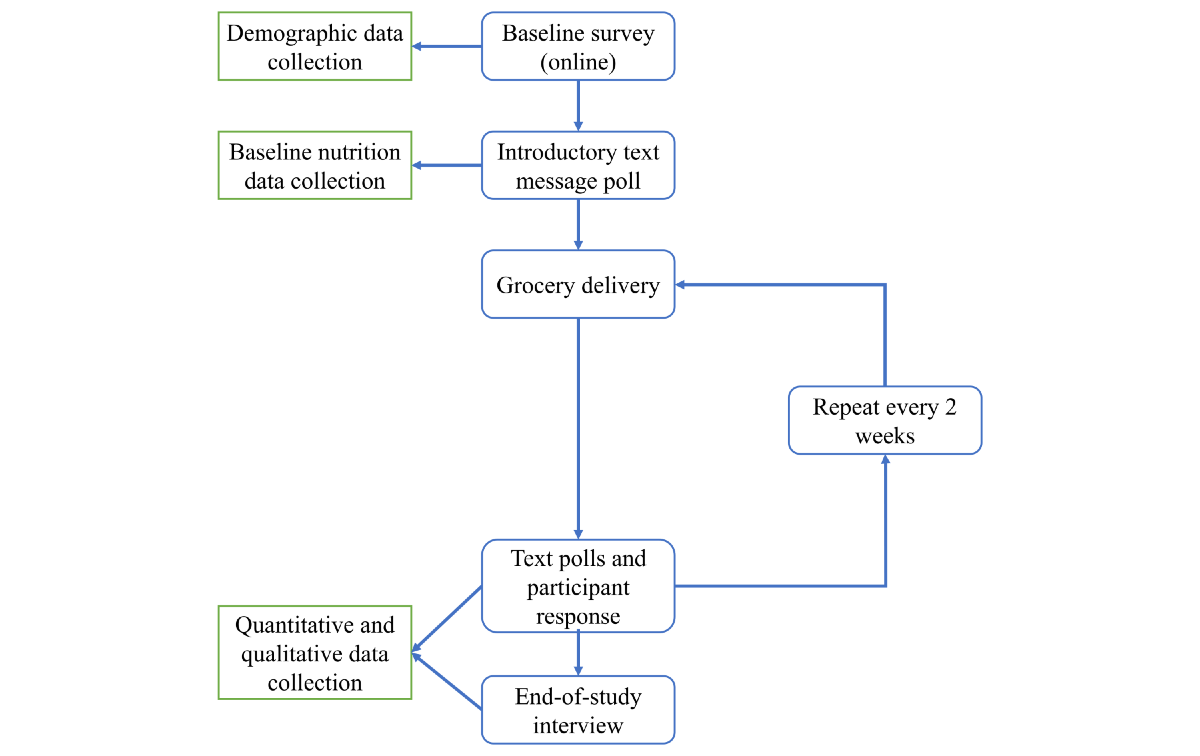
Outline of the special delivery process.

Special Delivery is a grocery delivery program that was offered to WIC enrollees who were youths to improve healthy weight gain during pregnancy. We leveraged a low-cost online service to provide healthy foods directly to youths' homes throughout their pregnancy. Data were collected by phone, primarily via text messaging, allowing for participants to quickly and easily contribute their perspectives in an accessible medium. During the study, real-time participant feedback was used to improve data collection tools and delivery processes. An outline of the Special Delivery study process is depicted in Figure 1. This study was approved by the University of Michigan institutional review board (HUM00140840).

### Recruitment

Youth were referred to Special Delivery by local WIC offices in Genesee, Washtenaw, and Wayne counties. Eligibility criteria were age 14-24 years, enrollment in WIC, first pregnancy, gestational age <24 weeks, low-risk singleton pregnancy, fluent in written and spoken English, access to a phone with text messaging capabilities, and home address within the radius of the delivery service. Low-risk pregnancy was determined based on self-reported absence of high-risk conditions such as pre-eclampsia, gestational diabetes, serious mental health conditions, or any other condition requiring specialty care. If youth were eligible, consent was obtained; parental consent was waived for minors as approved by the institutional review board both to protect the confidentiality of pregnant minors and because the study was of minimal risk to participants.

At enrollment, participants completed an intake assessment either online or over the phone with study coordinators to confirm study eligibility and collect participant demographic data including date of birth, phone number, home address, estimated date of delivery, prepregnancy weight, current weight, height, and socioeconomic variables. Food insecurity was assessed for all participants via a youth-validated 2-question scale [26,27]. All eligible individuals who completed the assessment online were called by a study coordinator to confirm participation, review study procedures, create a food delivery schedule, and collect baseline food preferences.

### Intervention Period

The intervention period of the Special Delivery program lasted from participant enrollment until infant delivery; depending on gestational age at enrollment, the intervention period ranged from 6-8 months. During the intervention, participants received twice-monthly grocery deliveries of healthy foods and daily text prompts for data collection. Participants were encouraged to send several types of text messages including photos to confirm grocery deliveries, photos of foods eaten by participants, and text responses to food frequency surveys. Phone and text message-based communication was used as it is low burden to youth, who commonly own mobile devices and communicate via text [28,29]. Textizen (Vox Metropolis Inc), a secure online platform with response-based automated texting that simulates an active conversation, was used to collect all text message data.

### Grocery Delivery

**Figure 2:**
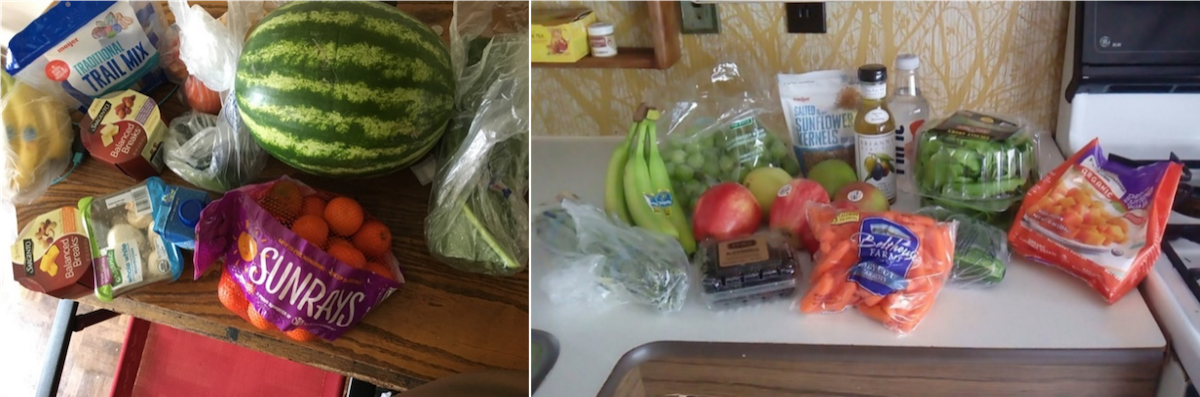
Example photos confirming receipt of typical grocery deliveries.

Twice-monthly grocery deliveries were made to Special Delivery participants until the end of their pregnancy (completion of the program) or unenrollment from the program. Each delivery contained US $35 worth of food and consisted of primarily WIC-approved foods for pregnant women such as seasonal fresh fruits and vegetables; frozen fruits and vegetables; and a variety of healthy snacks, including yogurt, cheese, whole grain cereal, whole grain waffles, popcorn, granola, coconut water, and flavored unsweetened water (examples are shown in Figure 2). Each delivery was approximately 75% fruits and vegetables with 3-4 healthy snack items. Participants were asked to share their food preferences at enrollment and again via text message 2 days prior to each grocery delivery. Deliveries were tailored to accommodate participant choice whenever possible.

Participants were asked to confirm receipt of each delivery by either texting a photo or calling the study coordinator. Specifically, confirmation could be completed by response to automated texts on the day of grocery delivery, response to automated texts on subsequent days before the next delivery, or directly with study coordinators by phone or text. Examples of photo confirmations submitted via text message are shown in Figure 2.

Groceries were delivered via the delivery service Shipt, a web- and app-based company that contracts with freelance shoppers to shop and deliver orders of groceries to home addresses. Shipt facilitates grocery delivery within a defined area (specified by zip codes) at any time the contracted grocery store is open for business. Deliveries are ordered for a specific timeframe and arrive within a 1-hour window of the selected time. The Special Delivery team managed its own Shipt account to coordinate deliveries and acted as the primary contact for questions related to substitutions or difficulties completing a delivery. This account was donated by Shipt for the purposes of this research project; however, a yearly subscription typically costs US $99. At enrollment, participants were asked to provide any details that could help Shipt shoppers in locating their address to successfully complete deliveries, including buzzer information, passcodes for gated communities, unofficial street names, or even colors of houses.

### Data Collection

To assess feasibility and acceptability of grocery delivery, daily data collection polls associated with each grocery delivery were sent via text message to participants and daily surveys prompted participants to confirm receipt of grocery deliveries and share examples of foods eaten to provide real-time data. Participants were encouraged to send photos of any foods consumed, regardless of whether they were from their grocery delivery. Additionally, all participants were contacted around their estimated infant due date for an end-of-study interview. Interviews were conducted over the phone by study coordinators in a semistructured fashion. Using an inductive approach, interview questions elicited feedback from participants about their perception of grocery deliveries, food quality, consumption of food sent, dietary impact of deliveries, and use of text message communication.

### Participant Incentives

Throughout the study, participants received incentives: US $10 for completion of the intake assessment, $10 for completion of end-of-study interviews, $1 for each day they responded to automated text surveys, and a $3 bonus for answering all text surveys in 1 month. Incentives were sent as Amazon gift cards via weblinks texted directly to the participants’ phones.

### Data Analysis

Using a mixed methods study design, we evaluated feasibility and acceptability with 2-way text message–based data collection and qualitative interviews (Table 1). Outcomes used to assess feasibility included quantification of the number of deliveries ordered by study coordinators, successfully delivered by Shipt shoppers, and confirmed by recipients. Acceptability outcomes were assessed by reviewing end-of-study interviews for perceptions of the grocery delivery process, delivered foods, and impact of grocery delivery on diet. Interview transcripts were reviewed by 2 researchers to identify themes in participant perceptions and feedback. Program acceptability was measured by frequency of positive perceptions reported by participants in interviews.

Participant food photos were analyzed by identifying the foods depicted in the image and categorizing them as either being consistent with groceries included in deliveries or not. A high frequency of foods that were consistent with foods in the deliveries was considered to be support for program acceptability.

**Table 1:** Program feasibility and acceptability measures.

Feasibility	Acceptability
Number of grocery orders placed	Participant perception of delivery process and delivered foods
Number of successful deliveries	Participant perception of impact on health
Number of confirmed deliveries	Foods eaten by participants

## Results

### Study Population

**Figure 3:**
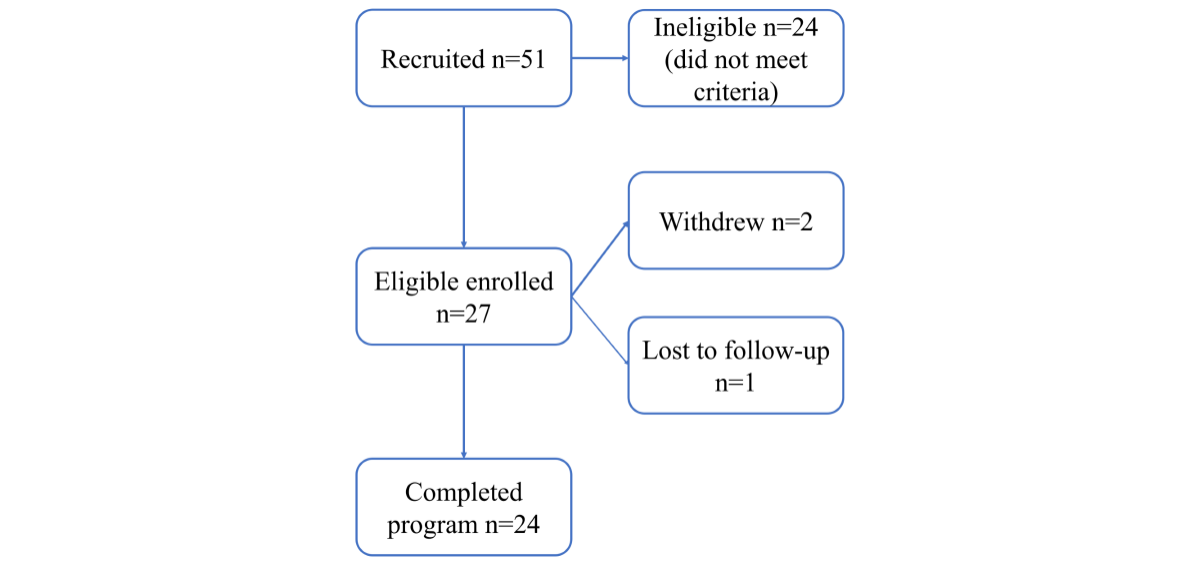
Participant enrollment and follow-up flowchart.

Enrollment in the Special Delivery pilot study ran from January 2019 to November 2019, and 27 participants were enrolled in the program, of which 24 participants completed their intervention periods as defined by the end of their pregnancy, and 21 completed the end-of-study interview (Figure 3). During the study, 1 participant did not confirm receipt of any grocery deliveries and was presumed lost to follow-up, resulting in stopped deliveries, 1 participant withdrew from enrollment prior to the end of her pregnancy because she did not have reliable access to an address for home delivery, and 1 participant withdrew from enrollment prior to the end of her pregnancy due to preference not to receive grocery deliveries. Participants who participated until their end of pregnancy received groceries for an average of 4.8 months (range 1.8-7.2).

Demographic characteristics of enrolled participants are displayed in Table 2. At enrollment, participants were between 17 to 23 years of age (mean 20.3, SD 2.0), and the mean gestational age was 16.3 weeks (SD 6.5). A majority of participants identified as non-Hispanic Black (16/27, 59%) and had attained a high-school level education or less (20/27, 74%); 56% of participants (15/27) reported a prepregnancy weight classified as normal (BMI between 18.5 and 24.9 kg/m^2^), and 30% of participants (8/27) reported BMI values classified as obese.

**Table 2:** Baseline demographic characteristics.

Characteristic			Value (n=27)
Age (years), mean (SD)			20.3 (2.0)
Gestational age at enrollment (weeks), mean (SD)			16.3 (6.5)
**Race/ethnicity, n (%)**			
	Non-Hispanic White	4 (15)	
	Non-Hispanic Black	16 (59)	
	Non-Hispanic other	5 (19)	
	Hispanic	2 (7)	
**BMI classification (kg/m^2^), n (%)**			
	Underweight (<18.5)	1 (4)	
	Normal weight (18.5-24.9)	15 (56)	
	Overweight (25-29.9)	3 (11)	
	Obese (>30.0)	8 (30)	
**Education level, n (%)**			
	Some high school	8 (30)	
	High school graduate	12 (44)	
	Some college	4 (15)	
	Associate’s degree	1 (4)	
	Bachelor’s degree	2 (7)	
**Food insecure (n=26), n (%)**			
	Yes	14 (54)	
	No	12 (46)	

### Program Feasibility

The Special Delivery program staff ordered a total of 263 deliveries during the pilot study. Of these, 259 deliveries (98.5%) of deliveries were considered successfully delivered. Only 1 delivery was reported as missing by the recipient and had to be reordered (<1%). In addition, 3 grocery orders (1.1%) were delivered later than the scheduled delivery hour, though they were delivered within 1 day.

Throughout the pilot study, Shipt shoppers communicated the outcome of every grocery delivery to study coordinators via text messaging. Shipt shoppers were self-directed and required relatively little contact from study coordinators to successfully complete grocery deliveries. Occasionally, Shipt shoppers were not able to hand off grocery deliveries directly to recipients because of difficulty locating addresses, restricted access to gated communities, or no answer at the front door. In these cases, Shipt shoppers discussed alternative delivery options with study coordinators, including asking for directions, leaving groceries at leasing offices or, as a last resort, at the front door of a house or apartment building. Study coordinators conveyed information between Shipt shoppers and the program participants receiving grocery deliveries.

**Figure 4:**
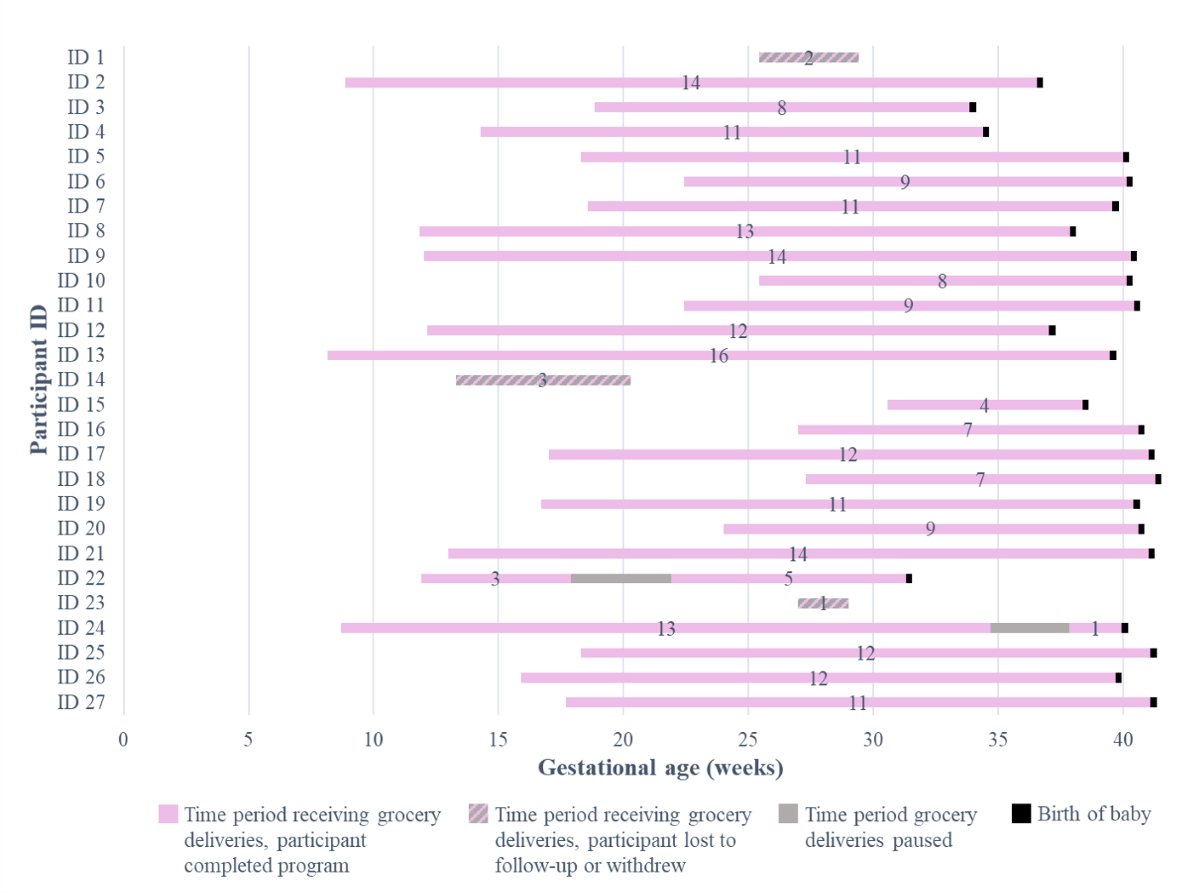
Each participant's intervention period with number of grocery deliveries received indicated.

Participants received deliveries twice monthly, and on average, participants received 9.7 deliveries in total (range 1-16 deliveries). The number of grocery orders delivered to each participant during the Special Delivery pilot is displayed in Figure 4. over the course of the pilot study, 1 participant paused deliveries for 4 weeks due to a shut-off in phone service (because no alternate contact method was listed) but was able to restore her phone service and continue to successfully receive and confirm grocery deliveries. In addition, 1 participant experienced an interruption in regular deliveries for three weeks due to limitations in the availability of grocery delivery services during the first few weeks of the Michigan stay-at-home order during the COVID-19 pandemic [30].

Participants confirmed receipt of 235 of the 263 deliveries (89.4%) by text, photo, or phone call. The low number of grocery deliveries reported missing by participants and the consistent communication from Shipt shoppers about the outcome of every grocery delivery both support the high fidelity of grocery delivery.

### Program Acceptability

Participants were asked about their perceptions of the Special Delivery process, including grocery delivery and the quality of delivered foods, during qualitative end-of-study interviews (Table 3). Nearly all participants who completed an end-of-study interview (19/21) reported that home delivery of groceries was convenient. Some participants also noted specific aspects of inconvenience (3/21). However, these were related to study procedures rather than the delivery of groceries (for example, 1 participant described the inconvenience of contacting study coordinators to change the day or time of delivery). Finally, 1 participant noted she felt that she had to change her clothes to be more presentable when answering her front door to receive her groceries.

Most participants (20/21) reported that delivered foods, including fruits and vegetables, were high quality, and 8 participants specifically described the freshness of fruits and vegetables as a positive aspect. Notably, 2 participants who reported foods as high quality also observed occasional instances when fruits and vegetables went bad within days of delivery, and 1 participant reported that the delivered foods were consistently low quality for the same reason. Despite these exceptions, perceptions of the Special Delivery process were strongly positive.

Participants were also asked about their perceptions of the impact of Special Delivery on health. Nearly all participants reported that the program helped them to have a healthy diet (20/21). Many participants reported improved access to healthy foods as a result of the Special Delivery program (15/21). In addition, most participants reported that the Special Delivery program helped them to build healthy habits (13/21). Examples of these healthy habits include substituting unhealthy food for healthy alternatives, cooking at home, tracking intake of healthy foods, maintaining adequate hydration, and shopping for more fruits and vegetables at the grocery store. Some participants also described trying new healthy foods as a result of the Special Delivery program (12/21).

All participants (21/21) recommended continuing or expanding the program to other pregnant women, and some even noted friends or family members who hoped to participate in a similar program. Not every participant gave an explanation about why the program should be continued, but many cited the positive impact on diet as a primary reason that other pregnant women could benefit from the program.

**Figure 5:**
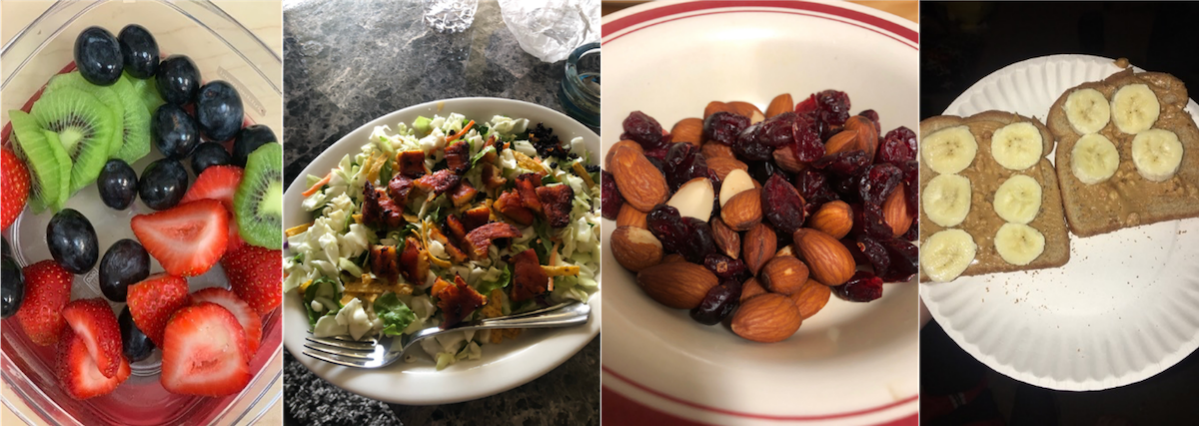
Example photos of delivered foods submitted by study participants in response to text message surveys.

Participants submitted a weekly average of 1.9 photos and 5.4 text messages during the program. Responses to daily text message polls included text descriptions as well as photos, which provided qualitative evidence of foods consumed during program participation (Figure 5). A majority of the photos that were submitted (591/821 71%) showed foods consistent with those included in grocery deliveries. This suggests that participants ate the delivered foods and supports the acceptability of the program.

**Table 3:** Categories of end-of-study interview feedback and representative quotations.

Categories		Representative quotations
**Delivery process**		
	Special Delivery is convenient	“It was convenient, like, you guys came when I actually needed something. And I liked it. I liked it a lot” “It’s just easy, someone comes to your door and then you have your groceries”
	Special Delivery foods were high-quality	“Everything that I got was fresh. I never got anything that was, you know, like spoiled or really close to the expiration date. So the shoppers did a really good job picking out, you know, the freshest food they could find”
**Impact on diet**		
	Special Delivery helped me have a healthy diet	“I would pack myself a lunch and most of it was from the delivery” “It kinda reminded me to eat more and eat healthier throughout the day” “Every time we got a salad, we ate those that day”
	Special Delivery improved my access to healthy foods	“Some days I wouldn’t eat at all, but since I had groceries in the fridge, like fruit, vegetables, I would just grab like some and take it for a snack” “It was just more healthy options in the house” “Being able to actually have access…just made it that much easier”
	Special Delivery helped me build healthy habits	“With this, it was like okay, I’m craving junk food, but maybe I’ll have an apple or an orange” “A lot of the things that I got were things that I don’t usually get at the store. So now when I go grocery shopping, those are things that I’m picking up”
	Special Delivery helped me try new foods	“I was able to try different stuff that I don’t normally get” “Let me just try it, let me eat it, so it don’t go to waste”
	Special Delivery could help other pregnant women	“It made me eat healthy stuff, so I guess it can make somebody else eat some healthy stuff too” “I think it’s good for all pregnant women” “It’s a little hard when you’re craving something bad for you. But just, just having that convenience is very helpful. So I think I would definitely continue it and have other moms experience it as well”

## Discussion

### Principal Results

Grocery delivery represents a well-established and inexpensive service that removes logistical barriers to obtaining healthy food but is underused by those who may need it most—young pregnant women. In this Special Delivery pilot study, we demonstrated the feasibility and acceptability of a grocery delivery–based weight gain and nutrition intervention among pregnant youth with low incomes. Grocery deliveries were successfully completed and confirmed by participants. Participants reported strong positive perceptions of the grocery delivery process and of the impact of delivered foods on health. Qualitative photo evidence submitted by participants demonstrated foods consistent with the healthy foods that were delivered. These results suggest the feasibility and acceptability of the Special Delivery program; participants were willing and able to receive grocery deliveries and eat the healthy foods that were delivered.

### Comparison With Prior Work

Our finding that grocery orders can be placed and delivered is consistent with prior studies [31,32] that demonstrated the feasibility of 1-time grocery delivery for residents of urban food deserts and of online grocery shopping for individuals with low income receiving food stamps. Though we limited inclusion to those residing in the delivery region of a single grocery delivery service (Shipt), the feasibility of this intervention could likely be extrapolated to most geographic areas since grocery delivery services continue to expand. Interview feedback and photos of the foods that were eaten demonstrated the high acceptability of grocery delivery to our participant cohort. Participants reported that the grocery delivery service was easy to utilize, convenient, and helped improve their diet quality, which is consistent with the findings of related studies of grocery delivery implementations as nutrition interventions [33]. Our acceptability findings differ from those from older studies [21,34,35] that concerns about the freshness of perishable goods were a major barrier to online grocery shopping acceptability for low-income populations. Instead, nearly all participants in our study reported that high-quality perishable foods and fresh fruits and vegetables were delivered.

The finding of acceptability from our pilot was also consistent with recent findings that a majority of grocery shoppers would be willing to order groceries online [36]. Our research provides new evidence that grocery delivery of healthy foods is a feasible and acceptable intervention for pregnant young women with low incomes.

### Limitations

Our assessment is not without limitations. We enrolled only a small cohort and have not yet explored the scalability of our methods. However, throughout our pilot, we focused on streamlining processes with the intention of creating a scalable method. For example, we now collect delivery instructions from participants during enrollment and provide participant contact information directly to Shipt deliverers to mimic real-life use of their service. Though participants did not order their own groceries, we are encouraged by the ease of the Shipt user interface and the potential for future participants to order on their own.

### Conclusions

Grocery delivery is an inexpensive service that has the potential to increase access to healthy foods for those who face significant logistical barriers to obtaining healthy foods. In our study, grocery delivery of healthy foods was found to be feasible and acceptable to pregnant young women. Our findings suggest that large-scale evaluation of the impact on gestational weight gain and overall quality of diet could further extend the potential benefits of grocery delivery to low-income young women and other vulnerable populations.

## Abbreviations

WIC: Special Supplemental Nutrition Program for Women, Infants, and Children
